# NSAIDs-hypersensitivity often induces a blended reaction pattern involving multiple organs

**DOI:** 10.1038/s41598-018-34668-1

**Published:** 2018-11-12

**Authors:** Inmaculada Doña, Esther Barrionuevo, María Salas, José Julio Laguna, José Agúndez, Elena García-Martín, Gádor Bogas, James Richard Perkins, José Antonio Cornejo-García, María José Torres

**Affiliations:** 1 Allergy Unit, IBIMA-Regional University Hospital of Malaga-UMA, Malaga, Spain; 2 ARADyAL network RD16/0006/0001, New York, USA; 3 Allergy Unit and Allergy-Anaesthesia Unit, Hospital Central Cruz Roja, Madrid, Spain; 4 ARADyAL network RD16/0006/0033, New York, USA; 5 Department of Pharmacology, University of Extremadura, Caceres, Spain; 6 ARADyAL network RD16/0006/0004, Cáceres, Spain; 7 Research Laboratory, IBIMA-Regional University Hospital of Malaga-UMA, Malaga, Spain

## Abstract

Non-steroidal anti-inflammatory drugs (NSAIDs)-induced hypersensitivity reactions are classified by the European Network on Drug Allergy (ENDA) as either cross-reactive or selective. The former is the most frequent type and includes patients with exclusively respiratory symptoms (NSAIDs-exacerbated respiratory disease, NERD) or exclusively cutaneous symptoms: NSAIDs-induced urticaria/angioedema (NIUA); and NSAIDs-exacerbated cutaneous disease (NECD). However, although not reflected in the current classification scheme (ENDA), in clinical practice a combination of both skin and respiratory symptoms or even other organs such as gastrointestinal tract symptoms (mixed or blended reactions) is frequently observed. This entity has not been sufficiently characterised. Our aim was to clinically characterize blended reactions to NSAIDs, comparing their clinical features with NERD and NIUA. We evaluated patients with symptoms suggestive of hypersensitivity to NSAIDs who attended the Allergy Unit of the Regional University Hospital of Malaga (Malaga, Spain) between 2008 and 2015. We included 880 patients confirmed as cross-reactive based on clinical history, positive nasal provocation test with lysine acetylsalicylate (NPT-LASA), and/or positive drug provocation test (DPT) with acetylsalicylic acid (ASA), who were classified as blended (261; 29.6%), NERD (108; 12.3%) or NIUA (511; 58.1%). We compared symptoms, drugs, underlying diseases and diagnostic methods within and between groups. Among blended patients the most common sub-group comprised those developing urticaria/angioedema plus rhinitis/asthma (n = 138), who had a higher percentage of underlying rhinitis (p < 0.0001) and asthma (p < 0.0001) than NIUA patients, showing similarities to NERD. These differences were not found in the sub-group of blended patients who developed such respiratory symptoms as glottis oedema; these were more similar to NIUA. The percentage of positive NPT-LASA was similar for blended (77%) and NERD groups (78.7%). We conclude that blended reactions are hypersensitivity reactions to NSAIDs affecting at least two organs. In addition to classical skin and respiratory involvement, in our population a number of patients also develop gastrointestinal symptoms. Given the high rate of positive responses to NPT-LASA in NERD as well as blended reactions, we suggest that all patients reporting respiratory symptoms, regardless of whether they have other associated symptoms, should be initially evaluated using NPT-LASA, which poses less risk than DPT.

## Introduction

Non-steroidal anti-inflammatory drugs (NSAIDs) are among the most frequent triggers of drug hypersensitivity reactions (DHRs)^1–4^. Several classifications have been proposed^5–8^, including a recently published and heavily cited publication from the European Network on Drug Allergy (ENDA) group from the European Academy of Allergy and Clinical Immunology (EAACI)^9^. They classify hypersensitivity reactions to NSAIDs according to the clinical symptoms induced, the number of NSAIDs involved, and the presence or absence of underlying diseases such as: (1) NSAIDs-exacerbated respiratory disease (NERD); (2) NSAIDs-exacerbated cutaneous disease (NECD); (3) NSAIDs-induced urticaria/angioedema (NIUA); (4) single-NSAID-induced urticaria/angioedema and anaphylaxis (SNIUAA), or (5) single-NSAID-induced delayed hypersensitivity reactions (SNIDHR). The first three groups are not thought to be immunologically-mediated, rather they are due to the inhibition of the cyclooxygenase (COX)-1 enzyme, with patients reacting to NSAIDs from chemically unrelated groups (cross-reactive hypersensitivity). The last two groups (SNIUAA and SNIDHR) encompass immunologically mediated reactions induced by a single NSAID/NSAIDs group (selective hypersensitivity)^9^.

Cross-reactive hypersensitivity to NSAIDs is the most frequent type of reaction for all age groups^10,11^. Classically, NERD is characterized by exacerbations of asthma and/or rhinitis after NSAIDs intake in patients with underlying chronic respiratory disease^12,13^. NIUA, the most frequent clinical entity^10^, is characterised by the acute appearance of cutaneous symptoms in otherwise healthy individuals, i.e. without chronic spontaneous urticaria (CSU)^9^. NECD occurs in patients with a history of CSU who develop an exacerbation of this pathology after taking NSAIDs. SNIUAA and SNIDHR are characterized by immediate or non-immediate reactions to a given NSAID or several NSAIDs from the same chemical group but tolerance to other non-chemically related NSAIDs^14,15^.

Although this classification is widely accepted and the groups outlined above represent well-defined phenotypes, not all clinical patterns fit neatly within a single category^5–8,10,16^. Patients with cross-reactive hypersensitivity to multiple NSAIDs may simultaneously develop a combination of skin and respiratory symptoms independently of the presence of underlying CSU, asthma or rhinosinusitis. For example, they often experience angioedema and/or urticaria alongside rhinoconjunctivitis and/or bronchial asthma after NSAIDs intake^5–8,10,16,17^. Such reactions involving at least two different organs were originally labelled as blended^6^, and in most cases the skin and airways are affected, although other organs may also be involved, such as the gastrointestinal tract^5–8,10,16,17^. However, in the ENDA classification, respiratory and cutaneous reactions are mutually exclusive and there is no single category in which to classify patients who have multiple organs affected after NSAIDs intake. Blended reactions have been estimated to account for more than a quarter of all reactions induced by cross-reactive hypersensitivity to NSAIDs in adults^10^ and up to 40% in children^17^.

To the best of our knowledge, no focused studies have been performed to evaluate the clinical characteristics and optimal diagnostic approaches for blended reactions, and it is still unclear whether these reactions are part of the NERD-NIUA spectrum, or a different entity altogether. In our clinical experience and in the literature^5–8,10,16,17^, blended reactions are almost never found in patients with NECD, as a result we excluded them from this study.

Here we have performed an in-depth analysis of patients suffering from blended reactions to NSAIDs, presenting some combination of cutaneous, respiratory and/or gastrointestinal symptoms. We have compared the clinical and demographic characteristics of these patients to those with exclusively respiratory (NERD) or cutaneous (NIUA) symptoms.

## Methods

### Patients

**Figure 1:**
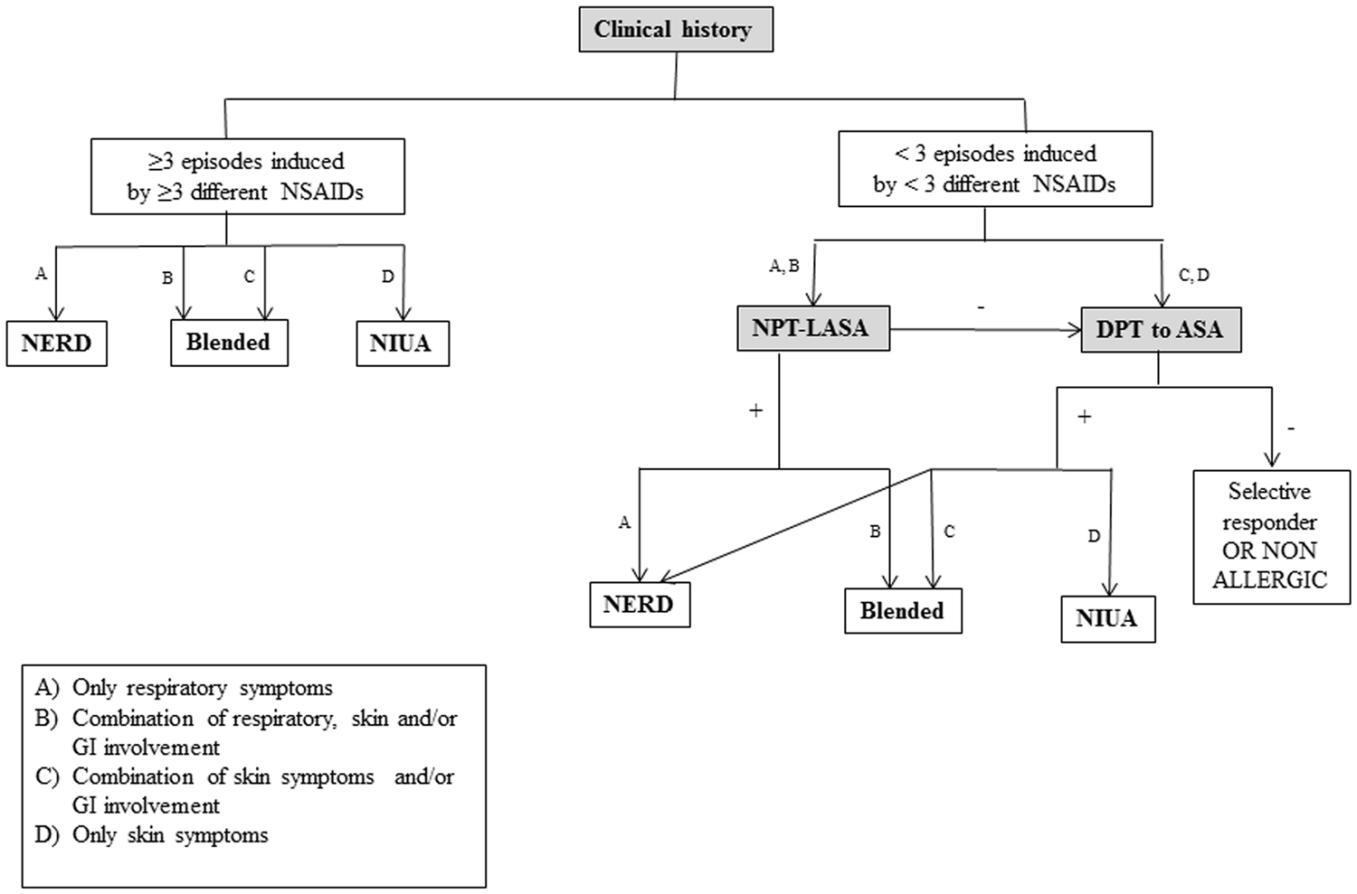
Algorithm for diagnosing patients with a history of hypersensitivity reaction after NSAIDs intake.

We evaluated patients aged 14–80 years old with symptoms suggestive of DHRs to one or more NSAIDs who attended the Allergy Unit of the Regional University Hospital of Malaga (Malaga, Spain) between 2008 and 2015. Of these, we included patients with a confirmed diagnosis of cross-reactive hypersensitivity to NSAIDs, defined by meeting at least one of the following criteria: (i) having experienced 3 or more episodes of cutaneous (urticaria and/or angioedema), respiratory (rhinitis, asthma and/or glottis oedema) and/or gastrointestinal symptoms (periumbilical colic pain, vomiting and/or diarrhoea) after the intake of at least 3 distinct NSAIDs from different chemical groups including a strong COX-1 inhibitor (acetylsalicylic acid (ASA) and/or indomethacin)^18^; (ii) having experienced less than 3 episodes of respiratory symptoms, with or without other organs involved after the intake of 2 different or only one NSAID, and giving a positive nasal provocation test with lysine acetyl salicylate (NPT-LASA); (iii) having had less than 3 episodes of cutaneous symptoms, with or without respiratory and/or gastrointestinal symptoms induced by less than 3 different NSAIDs and giving a positive NPT-LASA or drug provocation test (DPT) to ASA (Fig. 1).

Exclusion criteria included the following: patients with delayed DHRs such as fixed drug eruption, erythema multiforme, Stevens-Johnson syndrome, toxic epidermal necrolysis complex or acute generalised exanthematic pustulosis; pregnant or breastfeeding patients; patients taking beta-blockers or ACE inhibitors or with contraindications for epinephrine administration; patients with acute infections and/or underlying cardiac, hepatic or renal diseases that contraindicated DPT; patients with CSU that was exacerbated by NSAIDs (NECD); patients with psychosomatic disorders; patients reporting throat tightness not associated with dysphonia, difficulty breathing and swallowing and glottis oedema was not observed by fiberscope; and patients reporting gastrointestinal symptoms such as epigastric burning and hemorrhage related to alterations in the gastroduodenal mucosa secondary to the pharmacology action of NSAIDs.

The study was conducted according to the principles of the Declaration of Helsinki and approved by the Provincial Investigational Ethics Committee of Malaga. All participants were informed orally about the study and signed the corresponding informed consent. In the case of participants under the age of 18 years, informed consent was obtained from a parent and/or legal guardian.

### Patient classification

Patients were classified according to the symptoms experienced after ASA or other strong COX-1 inhibitor as: (i) NERD if they had respiratory symptoms (rhinitis and/or asthma); (ii) NIUA if they had skin symptoms (urticaria and/or angioedema); and (iii) blended if they had a combination of skin, respiratory and/or gastrointestinal symptoms (urticaria; angioedema; rhinitis; asthma; throat tightness associated with dysphonia, difficulty breathing and swallowing; periumbilical colic pain; vomiting and/or diarrhoea). Glottis oedema was observed using a fibroscope in patients reporting throat tightness associated with dysphonia, difficulty breathing and swallowing.

### Clinical history

Patients were questioned about the symptoms that appeared after NSAIDs intake, the time interval between drug intake and reaction onset, the number of episodes, the number of NSAIDs involved in the episodes, underlying nasal and bronchial symptoms (sneezing, itching, watery nose, nasal blockage, difficulty breathing, cough and wheezing), food allergy (urticaria, angioedema, oral allergy syndrome, anaphylaxis and shock), and CSU.

### Atopic status

Atopic status was assessed by skin prick test (SPT) using a panel of 20 common inhalant allergens, including pollens, house dust mites, moulds and animal dander, and 27 common food allergens including animal, fruit and vegetable allergens (ALK, Madrid, Spain). Histamine hydrochloride (10 mg/ml) and phenolated glycerol saline were used as positive and negative controls, respectively. Patients were requested to stop taking any antihistamine medication at least 8 days before undertaking SPT. A positive SPT response was defined as a wheal diameter of 3 mm or larger to at least one of these allergens and any patient producing such a wheal was considered atopic.

### Nasal and oral drug provocation testing

NPT-LASA was carried out as described in patients reporting respiratory symptoms regardless of the other organs involved^19^. Results were considered positive if an increase ≥30% in the total nasal symptoms and a decrease ≥30% in the total volume of both nasal cavities from 2 to 6 cm (vol 2–6 cm), measured by acoustic rhinometry, was observed.

Oral drug provocation testing (DPT) was performed in NIUA patients, and in blended and NERD patients who gave negative NPT-LASA results. DPT was performed in a single-blind manner as reported^10^. Briefly, placebo capsules were given at different times on the 1^st^ day; three doses of ASA were administered orally at intervals of 90 min (10, 50 and 50 mg) on the 2^nd^ day; and, if negative, three doses of ASA (125, 125 and 250 mg) were administered on the 3^rd^ day. If cutaneous and/or respiratory symptoms or changes in vital signs (rhythm alterations, decrease in FEV1 or hypotension) appeared, the procedure was stopped and symptoms were evaluated and treated. If no symptoms appeared during drug administration, the therapeutic dose of ASA was achieved and this was followed by a 2 day/8 h course at maximum dose, after a gap of 24 h. ASA and placebo were given in opaque capsules prepared by the hospital pharmacy service. Other medications were withheld before testing, according to international guidelines^20^.

### Statistical analysis

Descriptive statistics (frequency, mean, median and range) were used to present results, as indicated. Chi-square analysis was used to test differences for nominal variables, and the Fisher test was used when criteria for using the chi-square test was not met. For quantitative variables, non-parametric Mann-Whitney and Kruskal-Wallis tests were used. All reported p-values represented two-tailed tests, with values <0.05 considered significant.

## Results

A total of 2848 patients with symptoms suggestive of NSAIDs hypersensitivity were evaluated in the Allergy Unit of the Regional University Hospital of Malaga between 2008 and 2015. Of these, diagnosis could not be achieved for 1662 patients (1230 could not undergo DPT to ASA due to age or comorbidities, 376 refused to perform the study, and 56 were excluded due to pregnancy), and 267 individuals were diagnosed with selective reactions. This left a total of 919 patients (32.3%) diagnosed as having cross-reactive hypersensitivity. Of these, only 4.2% were classified as NECD, and were therefore not further considered.

Of the 880 patients finally included, 511 (55.1%) were diagnosed with NIUA, 261 (28.4%) classified as suffering blended reactions and 108 (11.7%) diagnosed with NERD. A total of 560 patients (63.6%) were female and the median age at diagnosis was 38 years (IQR: 28–50). Five hundred and eighty-one (66%) patients were atopic, the most common allergens being *Dermatophagoides pteronyssinuss* (359; 40.8%), *Olea europaea* (276; 31.4%), and *Lolium perenne* (192; 21.8%). A total of 420 (47.7%) had underlying rhinitis; 291 (33.1%) had asthma; 66 (7.5%) had nasosinusal polyposis and 15 (1.7%) had food allergy (4 to nuts, 9 to shellfish and 4 to melon).

A comparison of the demographic and clinical data between the different groups is shown in Table 1. We found that the proportions of patients having underlying rhinitis and asthma were higher in the blended group compared to NIUA (p = 0.0002 and p < 0.0001, respectively), but lower than NERD (p > 0.05 and p < 0.0001, respectively). In addition, polyposis was also less frequent in blended than NERD but more frequent than NIUA (p < 0.0001 for both comparisons). The proportion of atopic patients was similar when comparing blended with NIUA, but higher when comparing blended with NERD (p = 0.002) (Table 1 and Supplementary Table). No NIUA patient had nasosinusal polyposis and no patient diagnosed with NERD or as having blended reactions had food allergy.

**Table 1:** Demographic and clinical data of study participants.

	Blended n = 261	NERD n = 108	NIUA n = 511	p-value Blended vs NERD vs NIUA	p-value Blended vs NERD	p-value Blended vs NIUA	p-value NERD vs NIUA
Age median (IQR)	37 (29–48)	39 (30–48.5)	38 (28–50.7)	NS	NS	NS	NS
Gender (female), n (%)	176 (67.4)	73 (67.6)	296 (58)	0.03	NS	0.01	0.01
Underlying rhinitis, n (%)	158 (60.5)	77 (71.3)	185 (36.2)	<0.0001	NS	0.0002	<0.0001
Underlying asthma, n (%)	123 (47.1)	70 (64.8)	98 (19.2)	<0.0001	<0.0001	<0.0001	<0.0001
Nasosinusal polyposis, n (%)	24 (9.2)	42 (38.9)	0	<0.0001	<0.0001	<0.0001	<0.0001
Food allergy, n (%)	0	0	15 (3)	0.004	NS	0.005	NS
Atopy, n (%)	187 (71.6)	59 (54.6)	335 (65.5)	NS	0.002	NS	NS
Positive to at least one inhalant allergen, n (%)	181 (69.3)	59 (54.6)	328 (64.2)	NS	NS	NS	NS
Lolium;	91 (34.9)	19 (17.6)	82 (16)	0.001	0.03	0.0002	NS
Cupressus;	28 (10.7)	1 (0.9)	48 (9.4)	0.014	0.002	NS	0.005
Olea;	98 (37.5)	19 (17.6)	159 (31.1)	0.004	NS	NS	0.02
Parietaria;	19 (7.3)	9 (8.3)	30 (5.9)	NS	NS	NS	NS
Salsola;	22 (8.4)	2 (1.8)	40 (7.8)	NS	NS	NS	NS
D. pteronyssinuss;	108 (41.4)	32 (29.6)	219 (42.8)	NS	NS	NS	NS
Alternaria;	46 (17.6)	6 (5.5)	37 (7.2)	0.016	0.02	0.008	NS
Dog dander;	76 (29.1)	12 (11.1)	73 (14.3)	0.003	0.005	0.002	NS
Cat dander;	67 (25.7)	13 (12)	88 (17.2)	NS	0.03	NS	NS
Positive to at least one food allergen, n (%)	81 (31)	32 (29.6)	83 (16.2)	NS	NS	NS	0.02
Pru p 3	28 (10.7)	0	26 (5.1)	0.0001	0.0003	NS	0.01
Apple	24 (9.2)	0	10 (3.8)	<0.0001	0.001	NS	NS
Peanut	49 (18.7)	15 (13.9)	14 (2.7)	0.03	NS	0.04	NS
Walzetnut	49 (18.7)	15 (13.9)	15 (2.9)	0.03	NS	0.04	NS
Melon	23 (8.8)	0	22 (4.3)	0.0009	0.001	NS	0.02
Shrimp	29 (11.1)	10 (9.2)	14 (2.7)	0.01	NS	0.04	0.04

IQR: interquartile range; NS: Not significant.

Analyses of patient reactions according to clinical records are shown in Tables 2 and 3. Patients reported a median of 3 episodes after NSAIDs intake, and a median of 2 different NSAIDs were involved, with no differences between groups. The median onset time interval after NSAIDs intake was 45 minutes when considering patients from all groups (IQR: 20–120). When comparing between groups, blended reactions and NERD showed similar onset times (median: 30 minutes; IQR: 20–90, and median: 30; IQR: 15–120, respectively). However, this interval was shorter in patients with blended reactions compared to NIUA (median: 60; IQR: 30–120) (p = 0.0003) (Table 2).

**Table 2:** Clinical characteristics of the reactions based on data reported by patients.

		Blended n = 261	NERD n = 108	NIUA n = 511	p-value Blended vs NERD vs NIUA	p-value Blended vs NERD	p-value Blended vs NIUA	p-value NERD vs NIUA
Time interval drug-reaction, median (IQR) (min)		30 (20–90)	30 (15–120)	60 (30–120)	0.001	NS	0.0003	NS
Drugs involved n, (%)	ASA	105 (39.9)	34 (31.5)	194 (38)	NS	NS	NS	NS
	Indomethacin	3 (1.1)	—	7 (1.4)	NS	NS	NS	NS
	Diclofenac	59 (22.6)	20 (18.5)	106 (20.7)	NS	NS	NS	NS
	Ibuprofen	142 (54.4)	66 (61.1)	337 (65.7)	NS	NS	NS	NS
	Naproxen	18 (6.9)	6 (5.5)	36 (34.8)	NS	NS	NS	NS
	Dexketoprofen	18 (6.9)	8 (7.4)	44 (8.5)	NS	NS	NS	NS
	Dipyrone	108 (41.4)	30 (27.8)	205 (40)	0.004	0.004	NS	0.03
	Piroxicam	9 (3.4)	3 (2.8)	16 (3.1)	NS	NS	NS	NS
	Paracetamol	52 (19.9)	15 (13.9)	141 (27.5)	0.003	NS	NS	0.002
	Meloxicam	2 (0.8)	1 (0.9)	2 (0.4)	NS	NS	NS	NS
	Lysine clonixinate	7 (2.7)	2 (1.8)	9 (1.8)	NS	NS	NS	NS
	Selective COX-2	3 (1.1)	—	6 (1.2)	NS	NS	NS	NS
Number of episodes, median (IQR)		3 (2–4)	3 (2–4)	3 (3–4)	NS	NS	NS	NS
Number of drugs involved, median (IQR)		3 (1.25–3)	2 (1–3)	2 (2–3)	NS	NS	NS	NS
Time interval between the last reactions and diagnosis, median (IQR)		7 (3–24)	7 (5.5–8.5)	5 (2–23)	NS	NS	NS	NS

IQR: interquartile range; NS: Non significant.

**Table 3:** Symptoms induced by NSAIDs according to patient report.

Group	Symptoms		n (%)
Blended n = 261	Skin + Rhinitis/Asthma (Sub-group I) n = 138	AE + Asthma	48 (18.4)
		Urticaria + Asthma	48 (18.4)
		AE + Rhinitis	17 (6.5)
		Urticaria + AE + Asthma	8 (3.1)
		AE + Rhinitis + Asthma	7 (2.7)
		Urticaria + AE + Rhinitis + Asthma	5 (1.9)
		Urticaria + Rhinitis + Asthma	3 (1.1)
		Urticaria + AE + Rhinitis	2 (0.8)
	Skin + GE (Sub-group II) n = 100	AE + GE	59 (22.6)
		Urticaria + AE + GE	24 (9.2)
		Urticaria + GE	17 (6.5)
	Skin + Rhinitis/Asthma + GE (Sub-group III) n = 15	Urticaria + Asthma + GE	5 (1.9)
		AE + Rhinitis + GE	4 (1.5)
		AE + Asthma + GE	4 (1.5)
		Urticaria + Rhinitis + Ashtma + GE	2 (0.8)
	GI + Skin/Rhinitis/Asthma/GE (Sub-group IV) n = 8	GI + Rhinitis + Asthma	4 (1.5)
		GI + AE + Asthma	4 (1.5)
NERD n = 108	Rhinitis		20 (18.5)
	Asthma		64 (59.2)
	Rhinitis + Asthma		24 (22.2)
NIUA n = 511	Urticaria		89 (17.4)
	AE		124 (24.3)
	Urticaria + Angioedema		298 (58.3)

AE: Angioedema; GE: Glottis oedema; GI: Gastrointestinal symptoms.

Considering all groups together, 545 patients (61.9%) reported reactions to ibuprofen; 343 to dipyrone (39%); 333 to ASA (37.8%); 185 to diclofenac (21%); 208 to paracetamol (23.6%); 70 to dexketoprofen (7.9%); 60 to naproxen (6.8%); 28 to piroxicam (3.2%); 18 to lysine clonixinate (2%); 10 to indomethacine (1.1%), 9 to a selective COX-2 inhibitor (1%) and 5 to meloxicam (0.6%).

The percentage of reactions induced by dipyrone was higher in blended reactions compared to NERD (41.4% and 27.8%, p = 0.004). It was also higher for NIUA then NERD (40% and 27.8%, p = 0.03), as was paracetamol (27.5% vs 13.9%, p = 0.02). No significant differences found for other drugs (Table 2).

**Figure 2:**
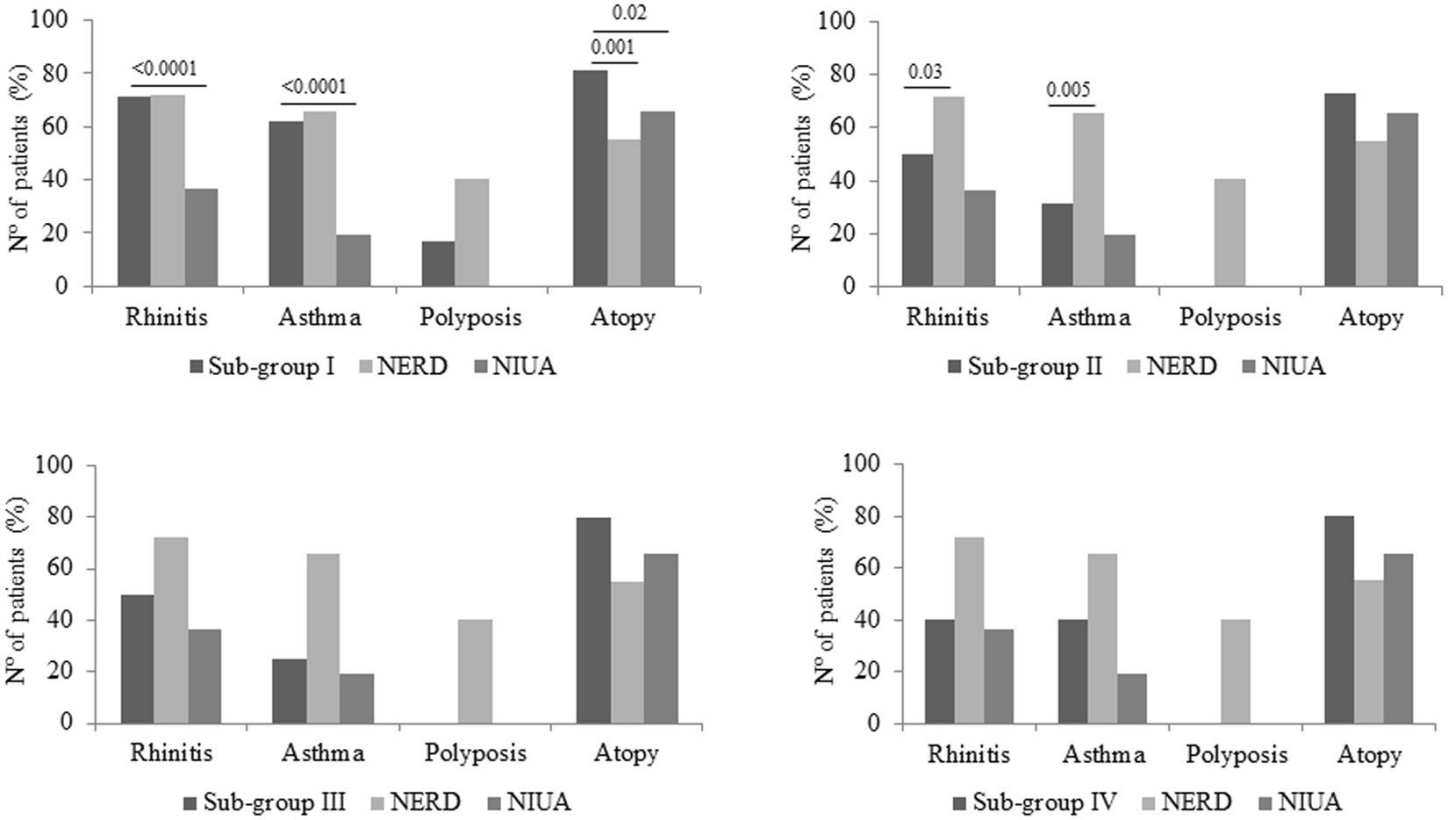
Percentage of underlying rhinitis, asthma, nasosinusal polyposis and atopy in the 4 Sub-groups of patients developing blended reactions (Sub-group I: Skin + Rhinitis/Asthma; Sub-group II: Skin + Glottis oedema; Sub-group III: Skin + Rhinitis/Asthma + Glottis oedema; Sub-group IV: Gastrointestinal symptoms + Skin/Rhinitis/Asthma/Glottis oedema) compared to NERD and NIUA.

Analysis of the clinical symptoms in the blended reactions group shows several general patterns (Table 3). Patients can be classified into 4 sub-groups: patients developing skin symptoms (urticaria/angioedema) and rhinitis/asthma (Sub-group I); patients developing skin symptoms (urticaria/angioedema) and glottis oedema (Sub-group II); patients developing skin symptoms (urticaria/angioedema), rhinitis/asthma and glottis oedema (Sub-group III); and patients experiencing a combination of gastrointestinal symptoms (abdominal pain, diarrhoea, nausea, vomiting) with skin symptoms (urticaria/angioedema) and/or rhinitis/asthma (Sub-group IV) (Table 3). Sub-group I was the most frequent (n = 138; 52.9%), followed by Sub-group II (n = 100; 38.3%), Sub-group III (n = 15; 5.7%) and Sub-group IV (n = 8; 3.1%). The proportion of underlying rhinitis, asthma, nasosinusal polyposis and atopy in the 4 Sub-groups of patients developing blended reactions is compared to NERD and NIUA in Fig. 2. Sub-group I contained a similar proportion of patients with underlying rhinitis (98; 71.01%) and asthma (85; 61.59%) to NERD patients (rhinitis: 77; 71.3%; asthma: 70; 64.8%), but different to NIUA (rhinitis: 185; 36.2%; asthma: 98; 19.2%) (p < 0.0001 for both comparisons). In Sub-groups II, III and IV, the proportions of patients with underlying rhinitis (Sub-group II: 50; 50%; Sub-group III: 7; 46.66%; and Sub-group IV: 3; 37.5%), asthma (Sub-group II: 31; 31%; Sub-group III: 4; 26.66%; and Sub-group IV: 3; 37.5%) and nasosinusal polyposis (zero for Sub-groups II, III and IV) were lower than in NERD, whereas underlying rhinitis and asthma showed similar proportions to NIUA. However, these differences were only significant for rhinitis and asthma between Sub-group II and NERD (p = 0.03 and p = 0.005, respectively). The percentage of atopy was similar for all subgroups of patients developing blended reactions, being similar to that of NIUA and higher than NERD, although significant differences were only found for Sub-group I, when compared to both NERD and NIUA (p = 0.001 and p = 0.02, respectively).

When considering the chronological appearance of clinical symptoms after NSAIDs intake by blended reaction patients, we found that 197 (75.5%) developed cutaneous symptoms first, followed by respiratory manifestations; 59 (22.6%) developed respiratory symptoms followed by skin and/or gastrointestinal symptoms, and 5 (1.9%) developed gastrointestinal symptoms followed by respiratory and/or skin symptoms.

The mean time interval between last reaction and diagnosis was 6 months (IQR: 2.5–24), with no statistically significant differences between groups.

The proportion of patients that could be diagnosed using only clinical history varied between groups (p < 0.0001). For NIUA it could be used for the diagnosis of 340 patients (66.5% of the total NIUA patients), for NERD it could be used for 22 (20.4% of the total NERD patients) and for blended it was 41 (15.7% of the total blended patients). In 286 patients (32.5%) the diagnosis was confirmed by NPT-LASA: 85 from the total of NERD patients (78.7%) and 201 from the total of blended patients (77%) (Table 4). Considering the 4 sub-groups of patients with blended reactions mentioned above, no statistically significant differences were found in terms of the percentage of patients giving positive NPT-LASA: 84.8% for Sub-group I, 66% for Sub-group II, 80% for Sub-group III, and 75% for Sub-group IV. None of the patients who underwent NPT-LASA suffered bronchial symptoms or a significant fall in FEV1 upon administration.

**Table 4:** Methods used to achieve diagnosis for each clinical entity.

Group	Diagnosis method n (%)		
	Clinical history	NPT-LASA	Positive DPT to ASA
Blended n = 261	41 (15.7)	201 (77)	19 (7.3)
NERD n = 108	20 (18.54)	85 (78.7)	3 (2.8)
NIUA n = 511	340 (66.5)	—	172 (33.6)
p	<0.0001	NS	<0.0001

The proportion of patients that were diagnosed using DPT also varied between groups (p < 0.0001). For NIUA it could be used for the diagnosis of 172 patients (33.6% of the total NIUA patients), for NERD it could be used for 3 (2.7% of the total NERD patients), and for blended it was 19 (7.3% of the total blended patients). Patients reacted to a median cumulative ASA dose of 250 mg (IQR: 100–500) with a median time interval of 75 minutes (IQR: 30–120) after the last administered dose. Although no statistical differences were found, NERD patients tended to react to a lower ASA dose (median: 75 mg; IQR: 46.25–200) compared to NIUA (median: 300 mg; IQR: 100–500) and blended reaction patients (median: 250 mg; IQR: 113.75–500) (Table 5). Patients with positive DPT responses to ASA experienced similar symptoms to those recorded in their clinical history, however they were generally milder, disappearing within 1–2 h after taking corticosteroid and antihistamine drugs and, if bronchial symptoms occurred, inhaled salbutamol. None of the patients required adrenaline to resolve their reaction.

**Table 5:** Cumulative dose ASA and time interval between ASA administration and reaction in positive DPT.

Group	Cumulative dose ASA in positive DPT, median (IQR)	Interval (minutes) ASA dose-positive response in DPT, median (IQR)
Blended n = 261	250 (113.7–500)	60 (30–112.5)
NERD n = 108	75 (46.2–200)	60 (51.2–75)
NIUA n = 511	300 (100–500)	90 (30–165)
p	0.4002	0.6519

IQR: interquartile range.

## Discussion

Not all entities induced by hypersensitivity to NSAIDs fit neatly within the ENDA classification^9^. For example, patients often develop cutaneous and respiratory symptoms simultaneously^5–8,10,16^. By evaluating a large series of patients with cross-reactive hypersensitivity to NSAIDs we found such blended reactions to be the second most frequent reaction type after NIUA in our population, representing more than 28% of cases in this study, in agreement with previous findings^10^.

The aim of this study was to characterize patients developing blended reactions and to compare them to those developing exclusively respiratory or cutaneous symptoms. In our population, no NECD patients experienced respiratory symptoms in combination with cutaneous symptoms after NSAIDs intake and no blended patients had underlying chronic urticaria. Therefore, we used NIUA patients to represent patients with exclusively cutaneous manifestations for comparisons purposes and NECD patients were not further considered.

Although it is thought that both NERD and NIUA may involve COX-1 inhibition, they are also thought to represent two distinctive phenotypes, as can be seen in the response to NPT-LASA and the release of inflammatory mediators^19,20^. In blended patients there is an overlap of clinical entities as they share the same NSAIDs-induced respiratory and cutaneous with NERD and NIUA, respectively. Moreover, blended patients have a similar proportion of underlying rhinitis than NERD and atopy than NIUA. However, although the proportion of underlying asthma and nasosinusal polyposis is higher than NIUA, is lower than NERD. Iit is unclear whether blended reactions represent a further phenotype or are part of a NERD-NIUA spectrum. This is especially important for those patients that develop throat tightness. Such tightness is considered as glottis oedema, a form of angioedema. As such, it should be decided whether these patients belong to the NIUA category or to NERD, as they have difficulty breathing due to upper airway involvement. Strikingly, we find that patients experiencing rhinitis and/or asthma accompanied with urticaria and/or angioedema have a similar clinical profile to NERD with respect to underlying diseases, and this is different from the profile of patients experiencing glottis oedema plus urticaria and/or angioedema, as well as NIUA patients. In addition, the percentage of positive responses to NPT-LASA in blended reactions is similar to NERD, with no significant differences between patients experiencing urticaria and/or angioedema accompanied by rhinitis and/or asthma and/or glottis oedema. Therefore, although patients with glottis oedema had a different clinical profile to NERD, appearing to be more similar to NIUA, the positive response to NPT-LASA allows it to be differentiated from NIUA. Previously, it has been reported that 12% of NIUA patients gave positive NPT-LASA^19^. However, these patients would be better classified as blended, as they reported both palpebral angioedema and glottis oedema after NSAIDs intake. Importantly, the NIUA patients in this study that gave negative NPT-LASA did not report glottis oedema or other respiratory symptoms in combination with skin symptoms. This has important implications for diagnosis, which we will discuss below.

We have also detected patients showing gastrointestinal plus respiratory involvement and/or skin symptoms. NSAIDs are known to induce alterations in the gastroduodenal tract due to direct action on the mucosa as well as prostaglandin synthesis. This can lead to a wide range of tissue damage from mucosal erosions to ulcus and perforation, inducing generally symptoms as epigastric burning and hemorrhage. The symptoms referred by our patients experiencing blended reactions are not related to such alterations in the gastroduodenal mucosa secondary to the action of NSAIDs, in our patients the gastrointestinal symptomatology is acute and includes periumbilical colic pain, vomiting and diarrhea, being related to the hypersensitivity induced by NSAIDs. In this sense, patients may be considered anaphylactic. Therefore, we suggest guidelines for NSAIDs hypersensitivity diagnoses and classification should take into account that cross-hypersensitivity reactions may have an anaphylactic component. Despite having a rather different clinical profile to NERD patients, these patients also gave a high percentage of positive response to NPT-LASA.

The high percentage of positive response to NPT-LASA in patients with blended reactions, especially in those that previously reported severe symptoms, is an important finding, with potentially important implications for diagnosis as this test is known to be safer than oral DPT^20^. Therefore, we suggest that NPT-LASA should be the first diagnostic approach in blended patients independently of the organ involved and we propose the inclusion of this test in the diagnostic algorithm for NSAIDs hypersensitivity reactions (Fig. 1).

Blended reactions represent a systemic clinical entity involving at least 2 organs, that can be severe and may be confused with SNIUAA. However, DPT with ASA can be used to differentiate between selective and cross-reactive hypersensitivity reactions^10,21,22^. This has important clinical implications, as patients with selective reactions can tolerate other NSAIDs^9^, whereas in cross-reactive hypersensitivity a safe alternative NSAID must be found.

Symptoms induced by ASA or other strong COX-1 inhibitors are crucial to define the phenotype, as the potency with which NSAID inhibit COX-1 can influence the symptoms induced by the drug. For example, meloxicam or paracetamol can induce cutaneous symptoms exclusively, whilst ASA induces skin and respiratory symptoms in the same patient. In our population, patients tended not to vary in terms of reported symptoms for repeated episodes, and for most of them a strong COX-1 inhibitor was involved. Patients with positive DPT responses to ASA experienced similar symptoms to those recorded in their clinical history, although generally milder, as they were challenged in a controlled manner and responded to lower doses. Only in 14 (5.36%) patients there was a difference in the phenotype established by clinical history (they reported only one episode which manifested as skin symptoms after paracetamol, meloxicam or etoricoxib intake and no strong COX-1 inhibitor was taken between the reaction and the study) compared to that established by DPT (skin and respiratory symptoms in DTP to ASA). Therefore, the phenotypes in our population were very reproducible.

Reports have shown that dipyrone and paracetamol are relatively safe drugs as therapeutic alternatives in NERD patients^23,24^ whereas up to 30% of subjects with NIUA may also be intolerant to these drugs^5,10,25,26^. Here, we found the number of NSAIDs involved and the percentage of patients having reactions induced by dipyrone and paracetamol to be higher in patients with blended reactions than for NERD. It would appear that patients developing blended reactions to NSAIDs show varying degrees of overlap with NERD and NIUA. Nevertheless, we do not know if NERD patients can develop skin symptoms over time, whilst long term studies of NIUA patients did not show the development of respiratory symptoms in the natural course of their disease^27,28^. Further longitudinal studies alongside investigation of the underlying mechanisms may help clarify the nature of the relationship between the different entities. Whilst the inhibition of COX-1 is thought to participate in these reactions^6,29^, and this has been studied in depth in NERD subjects^23^, additional studies are required to shed light on the mechanisms of NIUA and blended reactions.

Summarizing, the results of this study indicate that blended reactions are a frequently occurring, complex and potentially severe condition for which fewer alternative drugs may exist than for NERD, due to increased sensitivity to dipyrone and paracetamol. Given the frequency of these reactions, we would suggest further investigation with the aim of extending the ENDA guidelines in terms of including patients with blended reactions, with potential subdivisions within this category. We have also shown that NPT-LASA represents a useful diagnostic approach for these reactions and should be considered when dealing with a patient with blended reaction symptoms. Future studies should aim to clarify the pathomechanisms involved and the natural evolution of these reactions.

## Electronic supplementary material

Supplementary table
