# Supplementary material for: NSAIDs-hypersensitivity often induces a blended reaction pattern involving multiple organs
Source: Sci Rep. 2018 Nov 12;8:16710. doi: 10.1038/s41598-018-34668-1 (PMC6232098; doi:10.1038/s41598-018-34668-1)
Supplement: Supplementary file 1 — Supplementary table [file 41598_2018_34668_MOESM1_ESM.pdf]

TITLE: NSAIDs-hypersensitivity often induces a blended reaction pattern involving multiple organs.

AUTHORS: Inmaculada Doña[1,2]*, Esther Barrionuevo[1,2]*, María Salas[1,2], José Julio Laguna[3,4], José Agúndez[5,6], Elena García-Martín[5,6], Gádor Bogas[1], James Richard Perkins[7], José Antonio Cornejo-García[2,7#], María José Torres[1,2#].

AFFILIATIONS:

[1]Allergy Unit, IBIMA-Regional University Hospital of Malaga-UMA, Malaga, Spain.

[2]ARADyAL network RD16/0006/0001.

[3]Allergy Unit and Allergy-Anaesthesia Unit, Hospital Central Cruz Roja, Madrid, Spain.

[4]ARADyAL network RD16/0006/0033.

[5]Department of Pharmacology, University of Extremadura, Caceres, Spain.

[6] ARADyAL network RD16/0006/0004.

[7]Research Laboratory, IBIMA-Regional University Hospital of Malaga-UMA, Malaga, Spain.

*Both authors contributed equally to this work.

[#]Both authors contributed equally to this work.

Suplemmentary Table. Skin prick test results to a panel of inhalant and food allergens in study participants.

	Blended n=261	NERD n=108	NIUA n=511	p Blended vs NERD vs NIUA	p Blended vs NERD	p Blended vs NIUA	P NERD vs NIUA
**Positive to at least one inhalant allergen n (%)**	181 (69.3)	59 (54.6)	328 (64.2)	NS	NS	NS	NS
**Positive to at least one food allergen n (%)**	81 (31)	32 (29.6)	83 (16.2)	NS	NS	NS	0.02
Lolium; n (%)	91 (34.9)	19 (17.6)	82 (16)	0.001	0.03	0.0002	NS
Cupressus; n (%)	28 (10.7)	1 (0.9)	48 (9.4)	0.014	0.002	NS	0.005
Cynodon; n (%)	39 (14.9)	10 (9.2)	48 (9.4)	NS	NS	NS	NS
Olea; n (%)	98 (37.5)	19 (17.6)	159 (31.1)	NS	NS	NS	0.02
Platanus; n (%)	14 (5.4)	4 (3.7)	16 (3.1)	NS	NS	NS	NS
Parietaria; n (%)	19 (7.3)	9 (8.3)	30 (5.9)	NS	NS	NS	NS
Salsola; n (%)	22 (8.4)	2 (1.8)	40 (7.8)	NS	NS	NS	NS
Artemisa; n (%)	22 (8.4)	5 (4.6)	30 (5.9)	NS	NS	NS	NS
Betula; n (%)	1 (0.4)	1 (0.9)	5 (1)	NS	NS	NS	NS
Chenopodium; n (%)	25 (9.6)	8 (7.4)	49 (9.6)	NS	NS	NS	NS
Plantago; n (%)	22 (8.4)	6 (5.5)	42 (8.2)	NS	NS	NS	NS
D. pteronyssinuss; n (%)	108 (41.4)	32 (29.6)	219 (42.8)	NS	NS	NS	NS
D. farinae; n (%)	86 (32.9)	21 (19.4)	107 (20.9)	0.001	0.03	0.0001	NS
B. tropicalis; n (%)	60 (23)	27 (25)	117 (22.9)	NS	NS	NS	NS
L. destructor; n (%)	53 (20.3)	12 (11.1)	116 (22.7)	NS	NS	NS	0.03
Alternaria; n (%)	46 (17.6)	6 (5.5)	37 (7.2)	0.011	0.02	0.008	NS
Aspergillus; n (%)	3 (1.1)	4 (3.7)	3 (0.6)	NS	NS	NS	NS
Dog dander; n (%)	76 (29.1)	12 (11.1)	73 (14.3)	0.002	0.005	0.002	NS
Cat dander; n (%)	67 (25.7)	13 (12)	88 (17.2)	NS	0.03	NS	NS
Horse dander; n (%)	13 (5)	5 (4.8)	20 (3.9)	NS	NS	NS	NS
Avocado; n (%)	1 (0.4)	0	2 (0.4)	NS	NS	NS	NS
Almond; n (%)	17 (6.5)	0	14 (2.7)	0.002	0.006	NS	NS

Cashew; n (%)	0	0	0	NA	NA	NA	NA
Celery; n (%)	12 (4.6)	0	10 (1.9)	0.01	0.02	NS	NS
Pru p 3; n (%)	28 (10.7)	0	26 (5.1)	0.0001	0.0003	NS	0.01
Apple; n (%)	24 (9.2)	0	10 (3.8)	<0.0001	0.001	NS	NS
Cherry; n (%)	15 (5.7)	0	16 (3.1)	0.01	0.01	NS	NS
Hazelnut; n (%)	15 (5.7)	3 (2.8)	7 (1.4)	NS	NS	NS	NS
Peanut; n (%)	49 (18.8)	15 (13.9)	14 (2.7)	0.03	NS	0.04	NS
Walzetnut; n (%)	49 (18.8)	15 (13.9)	15 (2.9)	0.03	NS	0.04	NS
Sunflower seed; n (%)	1 (0.4)	0	15 (2.9)	0.01	NS	NS	NS
Cabbage; n (%)	15 (5.7)	0	20 (3.9)	0.03	0.01	NS	0.03
Melon; n (%)	23 (8.8)	0	22 (4.)	0.0009	0.001	NS	0.02
Tangerine; n (%)	12 (4.6)	0	7 (1.4)	0.003	0.02	NS	NS
Orange; n (%)	15 (5.7)	0	11 (2.1)	0.003	0.01	NS	NS
Kiwi; n (%)	17 (6.5)	0	11 (2.1)	0.0006	0.006	NS	NS
Pineapple; n (%)	1 (0.4)	0	1 (0.2)	NS	NS	NS	NS
Banana; n (%)	2 (0.8)	0	3 (0.6)	NS	NS	NS	NS
Gliadin; n (%)	0	0	10 (1.9)	NS	NS	NS	NS
Lettuce; n (%)	19 (7.3)	1 (0.9)	15 (2.9)	0.001	0.003	NS	NS
Bean; n (%)	12 (4.6)	0	7 (1.4)	0.003	0.02	NS	NS
Tomate; n (%)	3 (1.1)	0	9 (1.8)	NS	NS	NS	NS
Mustard; n (%)	12 (4.6)	0	11 (2.1)	0.02	0.02	NS	NS
Sesame; n (%)	0	0	7 (1.4)	NS	NS	NS	NS
Soy; n (%)	0	0	6 (1.2)	NS	NS	NS	NS
Wheat; n (%)	15 (5.7)	1 (0.9)	9 (1.8)	0.001	0.01	NS	NS
Shrimp; n (%)	29 (11.1)	10 (9.2)	14 (2.7)	0.01	NS	0.04	0.04

NS: Not significant; NA: Non applicable
